# Automated morphometry toolbox for analysis of microscopic model organisms using simple bright-field imaging

**DOI:** 10.1242/bio.037788

**Published:** 2019-02-27

**Authors:** Guanghui Liu, Fenfen Dong, Chuanhai Fu, Zachary J. Smith

**Affiliations:** 1 Department of Precision Machinery and Precision Instrumentation, University of Science and Technology of China, Hefei, Anhui 230027, China; 2 School of Life Sciences, University of Science and Technology of China, Hefei, Anhui 230027, China

**Keywords:** Fission yeast, Cell counting, Image segmentation, Morphology analysis

## Abstract

Model organisms with compact genomes, such as yeast and *Caenorhabditis* *elegans*, are particularly useful for understanding organism growth and life/cell cycle. Organism morphology is a critical parameter to measure in monitoring growth and stage in the life cycle. However, manual measurements are both time consuming and potentially inaccurate, due to variations among users and user fatigue. In this paper we present an automated method to segment bright-field images of fission yeast, budding yeast, and *C. elegans* roundworm, reporting a wide range of morphometric parameters, such as length, width, eccentricity, and others. Comparisons between automated and manual methods on fission yeast reveal good correlation in size values, with the 95% confidence interval lying between −0.8 and +0.6 μm in cell length, similar to the 95% confidence interval between two manual users. In a head-to-head comparison with other published algorithms on multiple datasets, our method achieves more accurate and robust results with substantially less computation time. We demonstrate the method's versatility on several model organisms, and demonstrate its utility through automated analysis of changes in fission yeast growth due to single kinase deletions. The algorithm has additionally been implemented as a stand-alone executable program to aid dissemination to other researchers.

## Abstract (precis)

**Summary:** We demonstrate a robust algorithm to count and extract morphology of model organisms, including shape-variant mutants, enabling measurements of large numbers of individuals without substantial time or labor.

## INTRODUCTION

Single-celled organisms at different cell-cycle stages display characteristic cell size and shape, and thus the morphology of the single-celled organisms is instrumental in determining the growth status of the cells. Similarly, the characteristic body plan of multicellular organisms reflects well the developmental stages of the organisms. For example, the Fission yeast *Schizosaccharomyces pombe*, a rod-shaped unicellular organism with a rigid cell wall, grows by tip extension and divides at a defined length of ∼14 µm (Chang and Martin, 2009). Intriguingly, mutant yeast cells bearing defective genes relevant to the cell cycle, cell polarity, the cytoskeleton and mitochondria often exhibit abnormal cell shapes: small, long, bent, and rounded, among others (Hayles et al., 2013). Thus, morphological measurements of fission yeast are a routine task in fission yeast studies. However, the shape of the cell primarily relies on inaccurate and time-consuming manual measurements. As studies increasingly evaluate large numbers of mutants, with each mutant requiring morphological measurements of 100s or 1000s of individual cells the need for a robust and automated algorithm for cell counting and morphological measurement is urgent. Budding yeasts and other model organisms such as *Caenorhabditis elegans* are similarly intensively studied thanks to their well understood genomes with sizes tractable for pan-genomic studies.

Automated algorithms for cell segmentation abound in the literature. In the past several years, several groups have published automated algorithms applied to budding yeast or other cells. Zhou et al. analyzed cell growth phase in HeLa cells through adaptive thresholding, morphological filtering, and a watershed segmentation process that involves merging over-segmented cell nuclei (Zhou et al., 2009). However, this method is primarily focused on fluorescent cell nuclei images, where, due to the separation between nuclei of neighboring cells, the segmentation task is relatively straightforward. Alanazi et al. demonstrated a simple maximum entropy-based thresholding followed by a watershed segmentation step that effectively segmented bacterial cells in images acquired by a quantitative phase microscope (QPM) (Alanazi et al., 2017). However, while the algorithm is simple, with a nearly 100% success rate, its performance depends critically on the flat background and minimal halo produced by the specialized QPM system. Van Valen et al. recently demonstrated the robust and adaptable use of convolutional neural networks for cell segmentation problems (Van Valen et al., 2016). Neural networks have previously been shown to yield excellent segmentation for a wide range of problems (Ronneberger et al., 2015; Kraus et al., 2015 preprint; Cireşan et al., 2013, 2012), but have not yet been applied to fission yeast. Furthermore, they require substantial training, where users must manually annotate images for hundreds of examples of each potential cell shape or cell type in order to achieve reliable performance (Sommer and Gerlich, 2013).

Results on yeasts have primarily focused on budding yeast, where the circular nature of the yeast is critical to the performance of the algorithms. For example, Kvarnstroem et al. used an innovative adaptive threshold to binarize yeast images, followed by a circular Hough transform to find each cell's center, and finally employing dynamic programming to extract cell contours (Kvarnström et al., 2008). However, through the use of the Hough transform, this method is exclusive to cells whose shape is highly circular. Versari et al. have also generated a complex algorithm for monitoring budding yeast over long time periods, rigorously benchmarking it against previous algorithms (Versari et al., 2017). However, as with the Kvarnstroem method, it is (and the algorithms it benchmarks against are) optimized for circular cells. Thus these methods have limited use beyond budding yeasts. Li et al. recently demonstrated that a simple segmentation of *S. pombe* is possible from a 34-image focal-stack of bright-field images taken by an automated microscope (Li et al., 2017). However, this pre-supposes an automated microscope, and obtaining the z-stack requires a substantial time investment per field-of-view. Their method also makes use of a solidity index (related to convexity of each cell) to separate cells from background objects, which, as we show below, is not valid for shape-variant cell mutants, or for larger organisms such as *C. elegans* where complex, noodle-like shapes yield low solidity values. Machine learning methods have been gainfully applied to yeast cell segmentation as well. Peng et al. developed PombeX, based on machine learning, to segment fission yeast images in different imaging conditions, such as differing illumination and focus conditions (Peng et al., 2013). Arteta et al. developed an algorithm termed CellDetect, biased on support vector machines (SVM) to correctly segment H&E-stained histology images, fluorescence images, and phase-contrast images (Arteta et al., 2012), and were shown to have reasonable performance on fission yeast images as well (Zhang et al., 2014). However, as with neural network approaches, this method requires manually annotated images to train the SVM framework.

Several studies have accurately profiled *C. elegans*, using complex and costly commercial software (Gosai et al., 2010), or self-developed code, including highly accurate worm segmentation using the free CellProfiler toolbox (Moy et al., 2009; Wählby et al., 2012). However, while these methods provide accurate image segmentation of both bright-field and fluorescence images, their performance on other model organisms has not been explored.

Considering that even single cells display a wide range of morphologies, ranging from roughly circular to rods to convex-curved or noodle-like structures, a simple and robust algorithm that can successfully segment bright-field images from non-convex cells without intensive manual image annotation is still an unmet need. In this paper we report a new algorithm based on marker-controlled watershed segmentation that effectively analyzes bright-field images of fission yeast, budding yeast, and *C. elegans*.

## RESULTS

### Segmentation of fission yeast, budding yeast and *C. elegans*

**Fig. 1.**
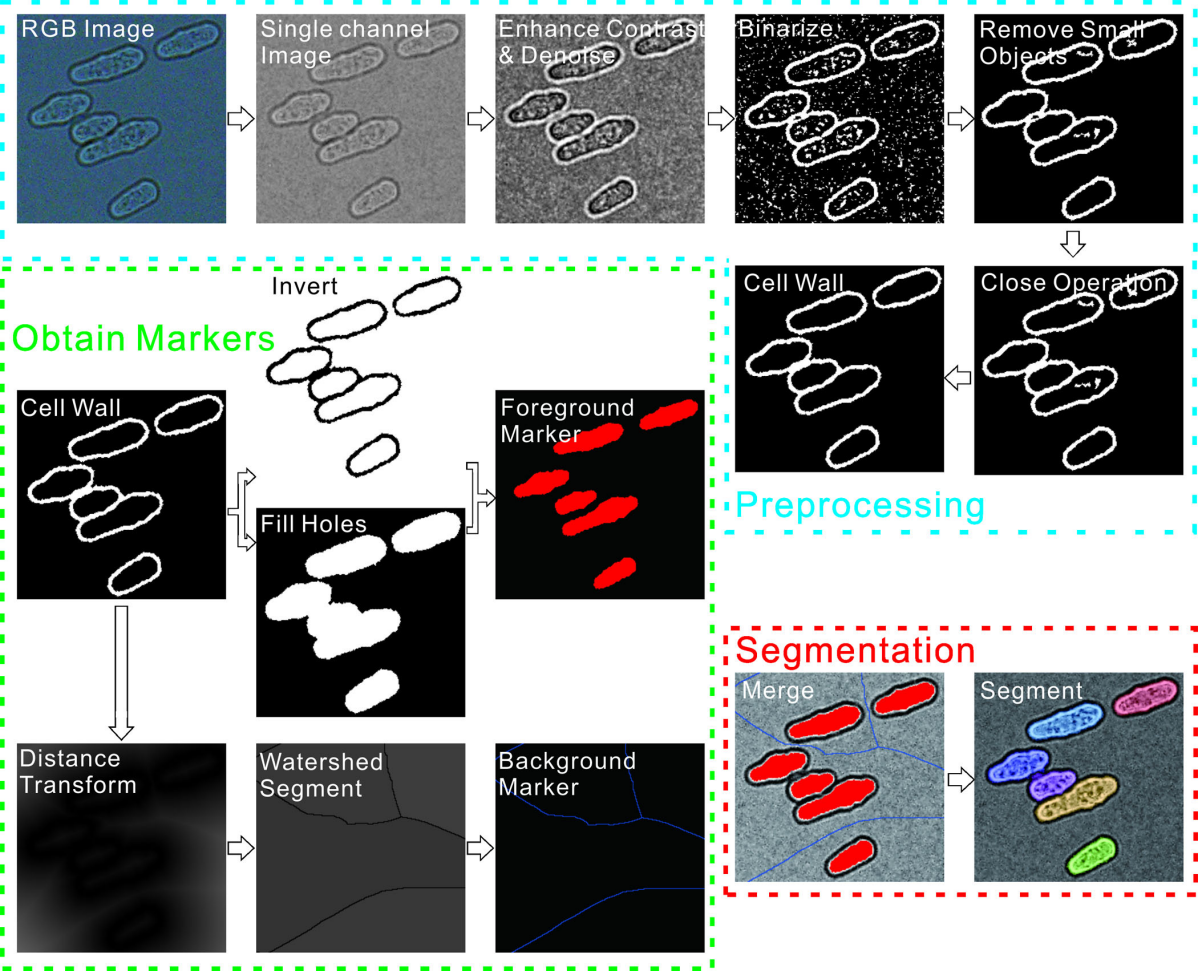
**Graphical depiction of the bright-field image algorithm.** The algorithm roughly includes three steps: preprocessing, foreground and background marking, and segmentation.

**Fig. 2.**
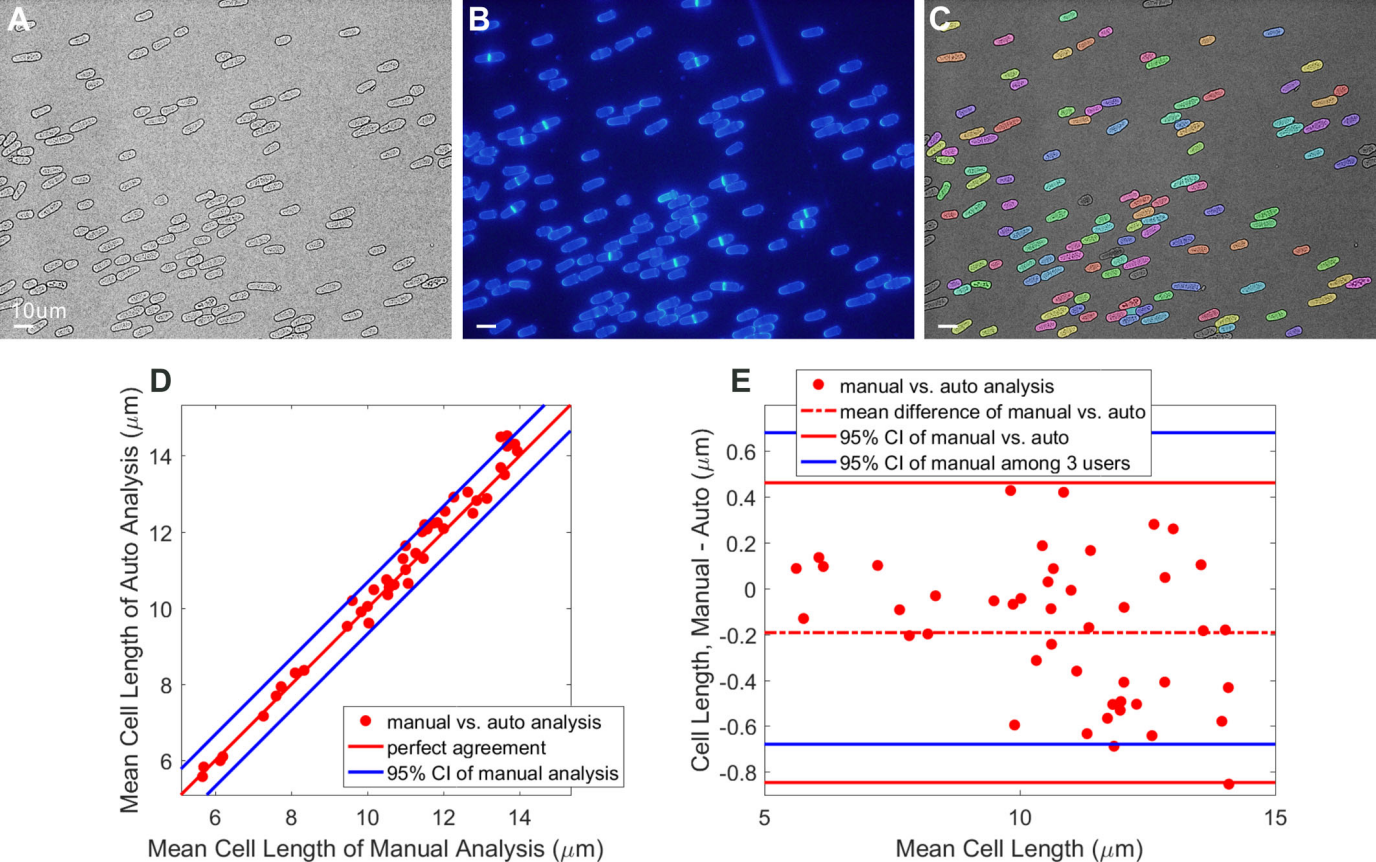
**Image data, algorithmic results, and** **comparison with manual analysis.** (A) Representative contrast-adjusted bright-field image of wild-type fission yeast cells. (B) Corresponding Calcofluor-White fluorescent image. (C) Final segmented result, where a false color overlay has been added to identify segmented regions. Note that same segmented image is also shown in Fig. 4A. (D) Mean cell length comparison between manual and automated analysis. Each dot represents mean cell length within one image averaged among three users versus mean cell length in that image via automated analysis, blue lines represent the 95% Conference Interval (CI) of manually-determined mean cell lengths among three different users. (E) Bland-Altman comparison between the average manually-determined cell length and the automated analysis. Each dot represents the mean cell length within a single image. Scale bars: 10 µm.

Our algorithm is optimized for bright-field images (graphical depiction in Fig. 1, example bright-field image shown in Fig. 2A), with an optional module, for the case of yeasts, to incorporate Calcofluor-White fluorescence information if available (example image shown in Fig. 2B, graphical depiction of algorithm shown in Fig. S1). While the results of the two algorithms are similar (Fig. S2), the fluorescence information increases the robustness of the algorithm to changes in focus or other user errors that may occur during standard image acquisition. Here we focus on the bright-field-only analysis, as this is the simplest to acquire experimentally.

Briefly, our approach to cell identification is marker-controlled watershed segmentation, where foreground and background markers aid traditional intensity-based watershed segmentation. As shown in Fig. 1, the algorithm roughly includes three steps, preprocessing, cell marking, and segmentation. The most critical step is obtaining foreground markers, which roughly identify each cell. Each cell should contain one and only one foreground mark. In order to obtain robust foreground markers, we use a logical procession of morphological processing steps. As described below, the steps are identical no matter which species we are segmenting, with the only variation being slight differences in image preprocessing and varying the exact size parameters inputted into the morphological operations, reflecting the varying size scales of the different model organisms.

**Fig. 3.**
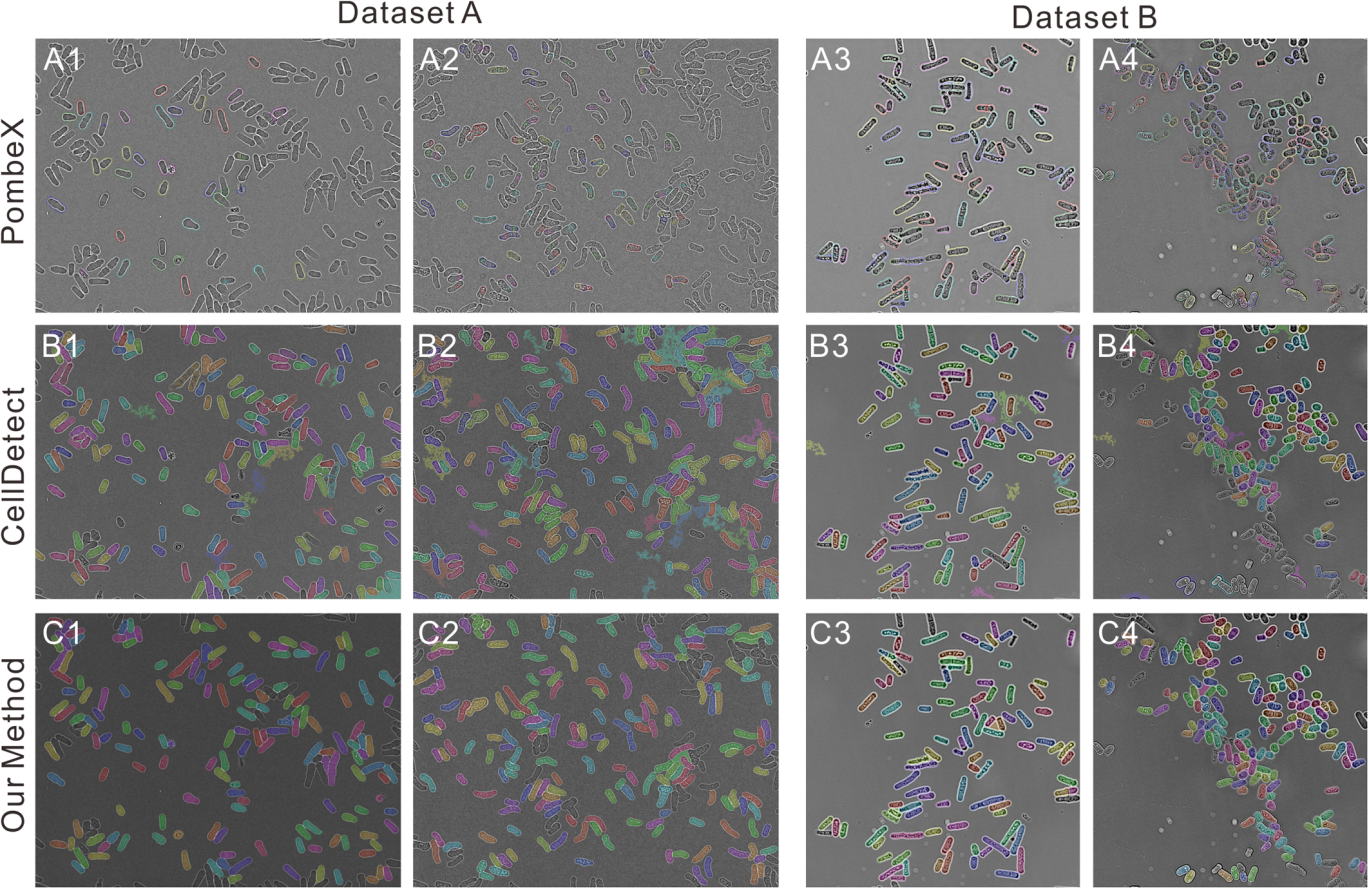
**Segmentation comparison between PombeX, CellDetect and our method by Dataset A from ours and Dataset B from the PombeX.** (A1-A4) Segmentation results by PombeX. (B1-B4) Segmentation results by CellDetect. (C1-C4) Segmentation results by our method.

As shown in Fig. 1, for fission yeast we start from a standard RGB bright-field image in the preprocessing step. Next we use the blue channel, which experimentally had the highest contrast, to perform the segmentation. We invert the single channel image such that the cell walls are bright against a dark background, adjust the contrast to more effectively use the dynamic range, and use a 2-D Gaussian smoothing kernel with standard deviation specified by 0.5 pixels to denoise the image. We then use Bradley and Roth's adaptive thresholding algorithm with a sensitivity scale of 35% (how different a pixel can be from its local mean to determine if it should be set to 1 or 0) to effectively binarize the image even under slightly non-uniform illumination (Bradley and Roth, 2007). Background objects with areas smaller than 150 pixels are removed. A morphological closing (dilation followed by erosion with a 2-pixel disk-shaped kernel) then ‘closes’ small gaps in the cell wall. This effectively creates a binary mask only containing the cell walls. Any cells touching the image boundary are removed. Using the cell wall image we begin to do the second step, obtaining markers. To mark the cell interiors (which will ultimately be our foreground markers), two copies of the cell wall mask are generated. In one, the holes in the image are filled, while the other is inverted. A logical AND operation is performed pixel-wise between the two images, yielding an image identifying only the ‘holes’ in the cell wall image, representing the cytoplasm. These are the foreground markers in our image. Background markers are produced through Skeleton by influence zones on the cell wall image (Vincent and Soille, 1991). The final watershed segmentation step is performed on the original contrast-adjusted and denoised bright-field image, with the image altered such that areas containing foreground and background markers are set to zero. The final segmentation result for a typical wild-type fission yeast image is shown both in Figs 1 and 2C. Notably, our algorithm can precisely segment images with crowded cells, as shown in Fig. 3. In later sections we demonstrate the effectiveness of our method despite the widely varying morphology of fission yeast mutants.

For the budding yeast segmentation, the procedure is identical to the fission yeast described above. However, after image inversion, we denoise the images using a total-variation constrained denoising algorithm (Smith et al., 2014). The morphological parameters are slightly altered to reflect the overall smaller size of the budding yeast cells. Furthermore, rather than using only the blue channel of the RGB image, the process is performed on both the green and blue channels separately. Once the foreground markers for each channel have been found, a logical OR operation is performed to combine the markers from both channels.

For *C. elegans* segmentation, an additional preprocessing step is included after inverting the image. Due to the large scale of the worm images, which lead to relatively non-uniform illumination (seen in Fig. 6A), a tophat operation is performed to ‘flatten’ the image background and remove the effect of the non-uniform illumination. Again, due to the varying scales between the yeast and worms, the parameters for the morphological operations are adjusted, but the order of operations remains the same, indicating the high degree of generalizability of our processing pipeline.

### Comparison between algorithmic counting and manual analysis

Once the cells are successfully identified using the algorithms described above, the morphology of each cell can be easily extracted from the result of the watershed segmentation. One of the most important morphological aspects of the cell is the cell length and cell width. These can be easily extracted from most yeasts by considering them as spheroids with a major and minor axis. To compare the performance of the cell size extractions of our algorithm, we obtained 46 images of wild-type fission yeast across 32 h (eight time points) in a nitrogen starve-release cycle to capture cells of highly varying sizes; the time points are nitrogen starve 0 h, starve 4 h, starve 8 h, and starve 24 h, followed by nitrogen release 2 h, release 4 h, release 6 h and release 8 h. Generally, yeast cells under nitrogen starvation undergo two rounds of continuous division within ∼7 h, causing the cell length to shorten (Yanagida et al., 2011). Cell length is lengthened again after medium replenishment. All the images were independently measured by both our automated algorithm and manual analysis by three users using MetaMorph (Molecular Devices, LLC. Sunnyvale, CA, USA).

The comparison of cell lengths between manual and automated analysis are shown in Fig. 2D, the x-axis represents the manually analyzed cell length, while the y-axis represents the automatically analyzed cell length. Each dot represents the mean cell length among all cells in a single image, and the manually analyzed cell lengths were further averaged among three users. As we can see from Fig. 2D, the mean manually-determined cell length and the automated results are quite similar. The correlation calculated between the mean manual and automated analysis is 0.9916, similar to the correlations between the three users (r=0.9884, see Table S1 for a complete tabulation of correlation values between different user groups). As the three manual users differ in their determination of cell length, we can compute the 95% confidence interval (CI) of agreement between each individual user and the mean manual analysis value, plotted as the blue lines in Fig. 2D. We can see that all the dots are located inside or on the blue lines, indicating that the difference between automated and manual analysis is similar to the likely disagreement between any two users.

To further compare the agreement of cell lengths analysis results between manual and automated methods, we performed a Bland-Altman analysis, as shown in Fig. 2E. The x-axis plots the mean of the automated and manual determinations, while the y-axis plots the difference between these methods. The dashed red line shows the bias between the automated and manual methods (−0.19 µm). The 95% confidence interval, shown with solid red lines in Fig. 1E, represents the maximum likely disagreement between the average manual and automated results. The 95% CI range between manual and automated analyses is −0.85 to +0.46 µm (range=1.31 µm), similar to the range of disagreement between the manual users (CI=−0.68 to +0.68 µm, range=1.36 µm). These results all demonstrate the reliability of the automated algorithm to robustly and reproducibly identify the cell lengths and widths, without the potential variability or bias introduced by manual counting performed by multiple users. Armed with this validation, we proceeded to compare the segmentation accuracy and time costs between the algorithm and two prior published methods.

### Comparison between different algorithms

To demonstrate the robustness and accuracy of our method, as well as its flexibility compared to previously reported methods, we compared our performance with two previously published algorithms for cell segmentation, PombeX and CellDetect. The segmentation is shown in Fig. 3, with the performance of each algorithm on each dataset summarized in Table 1. Accuracy was calculated as the number of correctly segmented cells divided by the total cell number. From Fig. 3 and Table 1, we can see that while PombeX performs reasonably well on Dataset B (for which it was trained), its robustness is extremely low, as when examining an unseen dataset, it achieves only 2% correct segmentation. If PombeX could be re-trained or have its parameters varied, it is possible that the segmentation rate in Dataset A could be improved. However, as currently available its performance is not satisfactory on datasets that differ in any way from the original data. Further, the output of PombeX does not yield any information about the cell morphometry. CellDetect, meanwhile, can be re-trained on a new dataset, although some manual effort must be expended to annotate the new images, and adjust their contrast to achieve optimum results. Following this training, the CellDetect algorithm can achieve reasonably good performance, around 65% correct segmentation on Dataset A and 76% on Dataset B. We note that Dataset A contains several shape-variant mutants, and the CellDetect algorithm's success was 84% on wild-type fission yeast. However, when analyzing yeasts with varying shapes, the performance markedly dropped. Including examples of these mutants in the training data had the effect of reducing overall performance. Further, due to the complexity of the algorithm, the training process takes one to several hours depending on the number of training images and their size. The analysis time per image in the testing phase takes 30 s to several minutes depending on the image size. By comparison, our proposed method yields higher accuracy on both datasets than prior algorithms, 85% and 90% correct segmentation respectively, with substantially faster computation time, approximately 5 s per image, and with higher length accuracy compared to manual analysis than CellDetect.

**Table 1.**
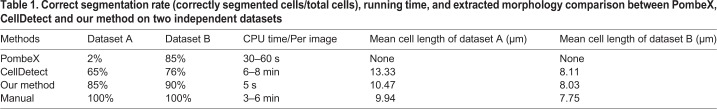
**Correct segmentation rate (correctly segmented cells/total cells), running time, and extracted morphology comparison between PombeX, CellDetect and our method on two independent datasets**

### Morphometric parameters for identification of fission yeast shape

**Fig. 4.**
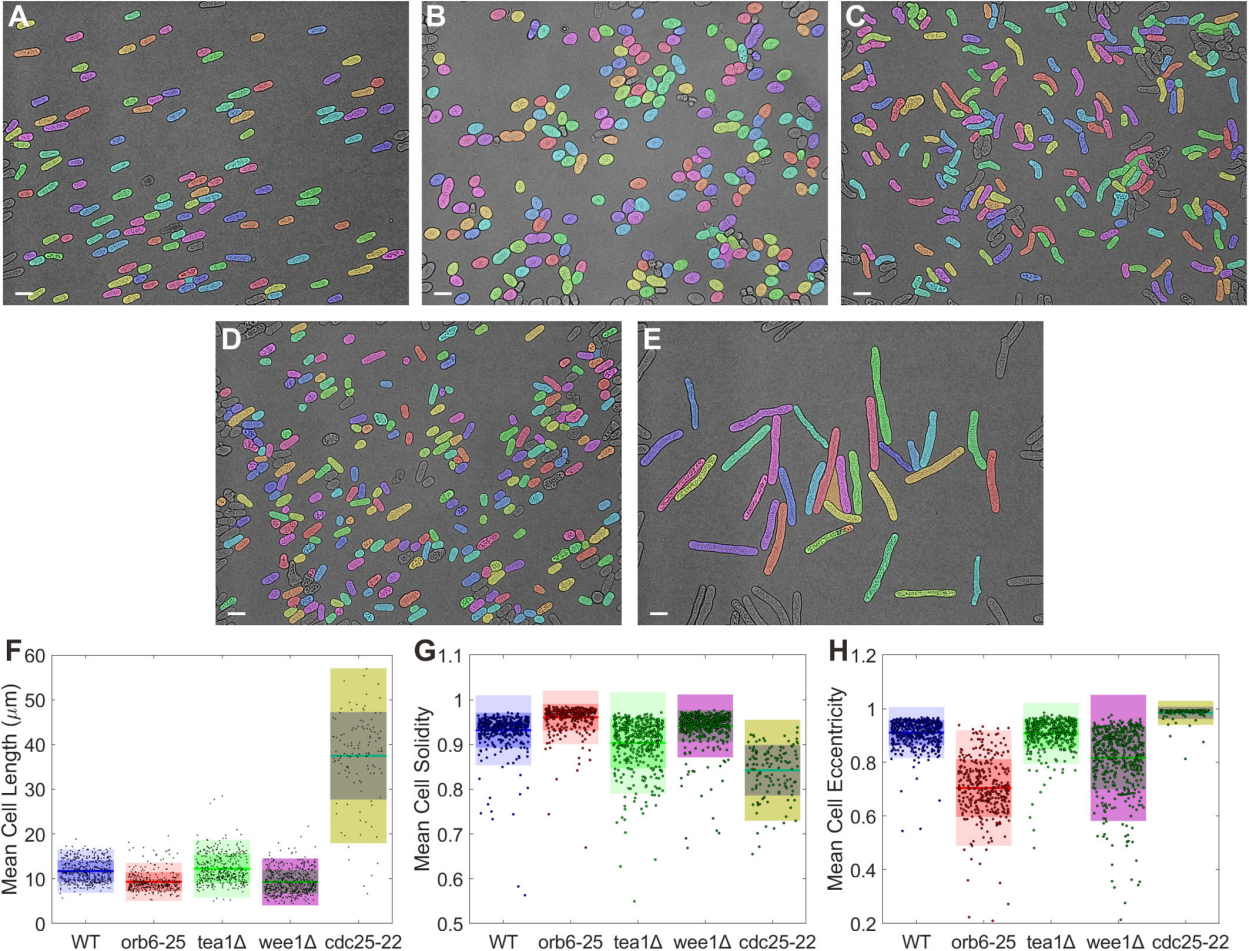
**Segmentation of wild-type and mutant fission yeast cells with altered shape.** (A) Wild-type cells. Note that the results in A are the same as shown in Fig. 2C. (B) orb6-25 temperature sensitive cells. Orb6 belongs to the conserved NDR family kinase required for polarized cell growth. (C) tea1Δ (tea1-deletion) cells. Tea1 localizes to cell ends to regulate cell growth, and the absence of Tea1 leads to bent and/or T-shaped cells. (D) wee1Δ cells. Wee1 is a kinase responsible for inactivating the master regulator Cdc2/CDK1 of the cell cycle. The absence of Wee1 lead to small cells. (E) cdc25-22 temperature sensitive cells. Cdc25 is a phosphatase responsible for inactivating Cdc2/CDK1. Inactivation of Cdc25 results in long cells. (F) Cell length, (G) solidity, and (H) eccentricity of five fission yeast strains, showing substantial variation among strains. Scale bars: 10 µm.

As discussed above, the algorithm, once it has correctly segmented the image, can easily extract a multitude of morphometric parameters. In addition to cell length and width, it can calculate simple metrics such as area or eccentricity, as well as complex measures such as the convex area, or measures of the image intensity within the cell. These can all be exploited to determine subtle differences in cell size and cell shape that are related to cell growth and function. To test the robustness of our algorithm, we analyzed several morphometric values for wild-type, orb6-25 (an orb6 temperature sensitive mutant; Orb6 is inactive at the restrictive temperature 37°C), tea1Δ (cells lacking Tea1), wee1Δ, and cdc25-22 fission yeasts. It has been reported that malfunction of Orb6 and Wee1 kinases leads to small round cells (Barbet and Carr, 1993; Das et al., 2009; Verde et al., 1998), while inactivation of Cdc25 makes cells longer (Keifenheim et al., 2017). The absence of Tea1, meanwhile, results in bent or T-shaped cells (Mata and Nurse, 1997). Representative segmentation results are shown in Fig. 4A–E, where the variegated morphology of the different strains can be readily observed. The results of automated morphometric analysis of these cells are shown in Fig. 4F–H and Table S2, where clear differences among the parameters between strains can be observed. For example, the mean eccentricity of orb6-25 and wee1Δ is lower than the other three strains, indicative of their circular nature. The ratio of the cell's area to the convex area (the area of the smallest convex polygon containing the cell), a measure of convexity called solidity, is smaller in tea1Δ and cdc25-22 than in other strains, showing the curved, concave nature of these cells. Meanwhile the mean length of wee1Δ is shorter than the wild type, while cdc25-22 is much longer. Altogether, our morphometric measurements recapitulate the results reported previously, confirming the accuracy of our method to segment yeast cells of widely varying shapes.

### Morphometric analysis of budding yeast

**Fig. 5.**
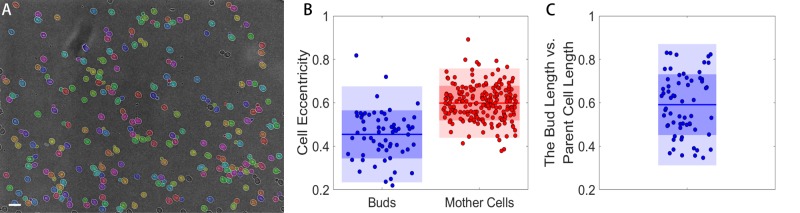
**Segmentation of budding yeast and morphologic analysis.** (A) Segmentation result of budding yeast. Scale bar: 10 µm. (B) Cell eccentricity comparison between buds and mature cells. (C) Length ratio between the buds and their parent cells.

For budding yeast, a critical point of interest is the morphometric relationship between daughter and parent cells. As shown in Fig. 5A, our algorithm can accurately identify the majority of buds in an image, despite their small size. Since the buds are smaller than mother yeast, we set an area threshold to separate the buds and mother yeast. Our automated analysis can also record the pixel locations of each identified cell in the image. To identify the most likely parent of a given bud, we find the closest cell to each bud and assign it as the bud's parent. As shown in Fig. S3, this accurately identifies the daughter–parent relationship in most cases. It is occasionally ‘fooled’ when a bud emerges in a crowded region and thus is touching multiple potential parents. With the bud and mother cells identified, their morphometry can be independently analyzed, as shown in Fig. 5B, where the bud’s eccentricity is smaller than the mother yeast, indicating that they are more circular compared to the slightly elongated mother cells. Knowing the daughter–parent relationship, we can compute the diameter ratio between the daughters and their parent cells as shown in Fig. 5C. The length ratio may be a key parameter to measure in studying cell size control as the daughter cell size is regulated in a cell-cycle dependent manner and by the availability of nutrients (Leitao and Kellogg, 2017).

### Morphometric analysis of *C. elegans*

**Fig. 6.**
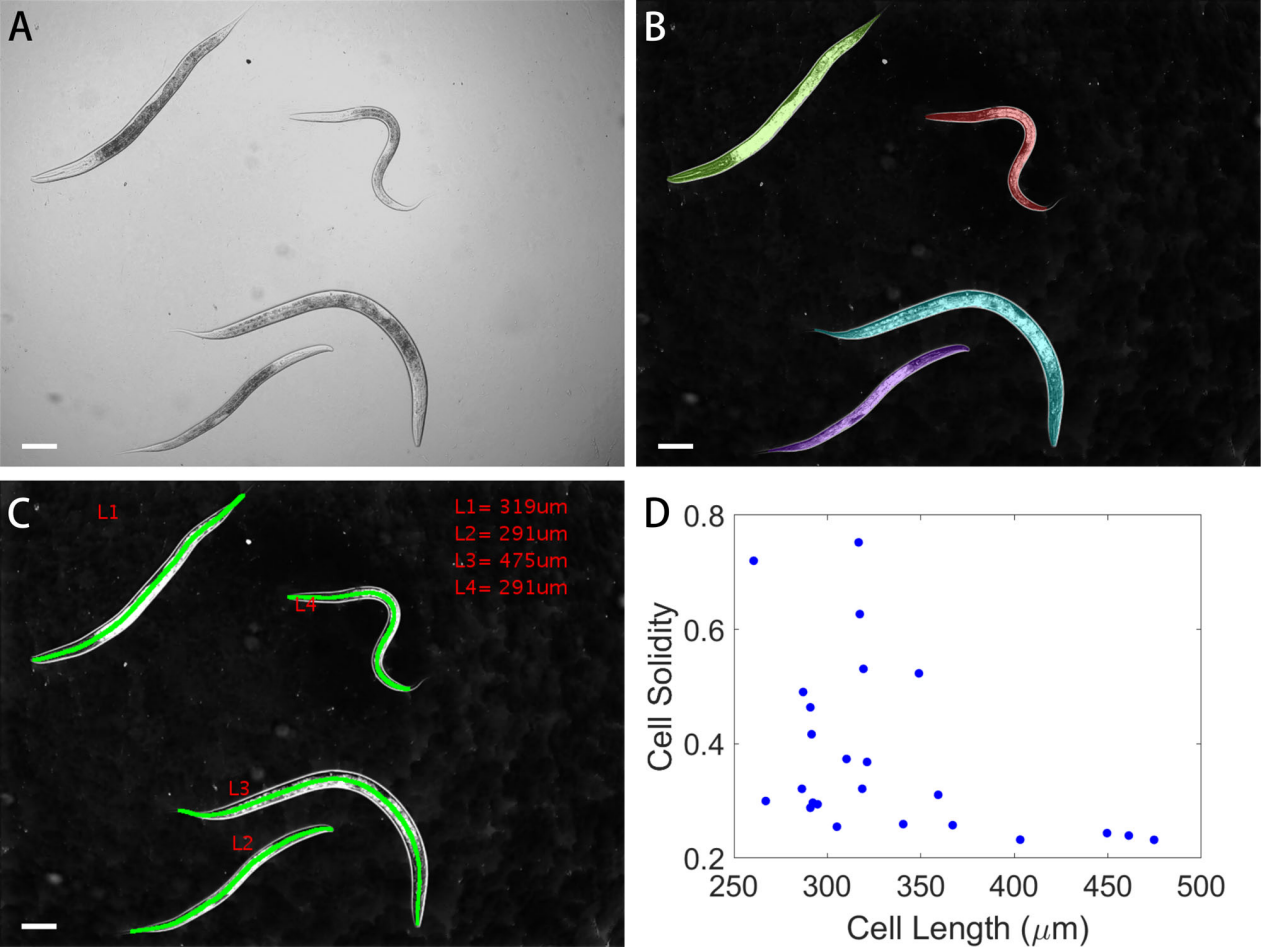
**Segmentation of *C. elegans* and morphologic analysis.** (A) *C. elegans* bright-field image. (B) Segmentation result of *C. elegans* overlaid on contrast-inverted bright-field image. (C) Results of automated length determination. (D) Cell length versus solidity of 24 worms. Scale bars: 50 µm.

For the *C. elegans*, the worm length is the most important indicator marking the developmental stage of the *C. elegans* is in the life cycle. Further, due to the worm's typically curved morphology during locomotion, straight worms are likely to be dead. These can be parameterized by the worm length and worm solidity (with dead worms having high solidity, curved/living worms having low solidity values). Despite the worm's highly curved nature, and despite its highly divergent size scale compared with the yeast cells, our algorithm can still effectively segment the worms even when imaged using large fields of view with poor illumination uniformity, as shown in Fig. 6A and B. Because the worm is highly curved, its length cannot be accurately judged by a measure along any single dimension of the segmented shape. To extract its length, we utilize a skeletonization procedure described graphically in Fig. S4. Briefly, we first skeletonize the image, yielding a single pixel-wide line with several branch and endpoints. The branched line can be split into several segments. If a segment contains an endpoint, it is considered an ‘end-segment’, if the segment merely connects two branch points we term this a ‘branch-segment’. This line is initially 8-connected. However, to unambiguously split this skeleton into branch- and end-segments, we minimally thicken the line to be 4-connected. Starting from each endpoint, we iteratively calculate the length of all paths from that endpoint to all other endpoints in the skeleton. This process is repeated until all possible end-to-end paths are explored, with the longest end-to-end path being judged as the worm's length, as shown in Fig. 6C. A comparison of extracted length and solidity parameters from 24 individual *C. elegans* worms are shown in Fig. 6D.

### Automated analysis of cell growth during nitrogen starvation and replenishment confirms that the TOR pathway is essential for cells to re-enter the cell cycle

**Fig. 7.**
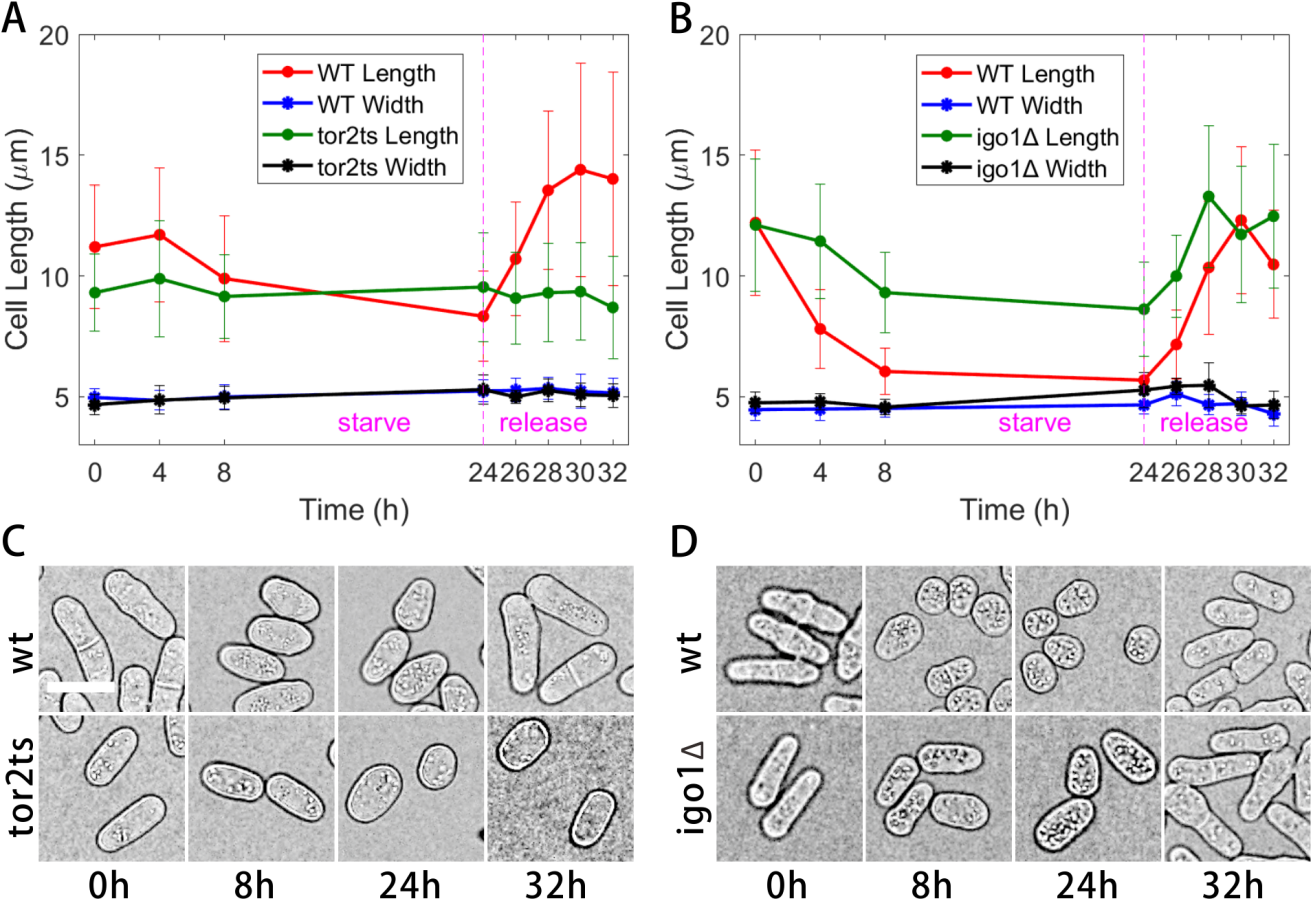
**Morphologic analysis of nitrogen starvation and replenishment of the indicated mutant fission yeast cells.** (A) Wild type versus tor2ts. (B) Wild type versus igo1Δ; error bars represent one standard deviation. (C,D) Contrast-adjusted bright-field image regions of interest comparing wild-type and mutant strains at the indicated time points: starve 0 h, 8 h, and 24 h, and release 8 h. Scale bar: 10 µm.

Because the algorithm presented above dramatically simplifies measurement of cell length, cell morphology can be easily observed in large numbers of cells over extended time periods with substantially reduced experimental effort. To demonstrate the power of such measurements, we followed wild-type and mutant fission yeast cells for 32 h of culture, including 24 h of nitrogen starvation followed by 8 h of release, as described in more detail in the Materials and Methods. In rich medium cells grow rapidly, and it is generally believed that the TOR pathway is involved in controlling such rapid cell growth by promoting anabolic processes and inhibiting catabolic processes. By contrast, cell growth ceases in medium lacking nitrogen due to the inactivation of the TOR kinase. In fission yeast, it has been demonstrated that tor2 (the TOR kinase TORC1) promotes cell growth by maintaining the activity of the phosphatase of PP2A.B55 through inhibiting the Greatwall(Ppk18)-Endosulfine(Igo1) pathway (Chica et al., 2016). Nitrogen depletion causes inactivation of the TOR signaling and then activation of the Greatwall(Ppk18)-Endosulfine(Igo1) pathway, inhibiting PP2A.B55 and enabling cells to enter mitosis prematurely with a smaller size (Chica et al., 2016). We then employed our automated algorithm to analyze the cell size of wild-type, Tor2 defective, and Igo1-deletion mutant cells during nitrogen starvation and replenishment. As expected, Tor2 was essential for initiating cell growth after nitrogen replenishment (i.e. re-entering the cell cycle) since cell size did not increase in cells without Tor2 activity (Fig. 7A,C). By contrast, the Tor2 downstream protein Igo1, also an inhibitory factor of PP2A.B55, appeared to be important for decreasing cell size (i.e. promoting premature mitosis) during nitrogen starvation but not important for the cells to re-enter the cell cycle during replenishment (Fig. 7B,D). Thus, our automated algorithm is a powerful tool to dissect cell growth and the cell cycle. The automated detection of multiple morphometric parameters also opens up the ability to track cell morphology in a multiparametric space, similar to flow cytometry, which may prove useful for identifying subtle effects or subgroups within a larger population (Fig. S5). Further, using the x and y positions of each cell in a time-lapse, single-cell growth curves such as shown in Mitchison and Nurse (1985), can be reproduced for wild type and shape-variant mutants (Fig. S6).

## DISCUSSION

In this paper we have presented a robust method to automatically segment images of yeast cells and *C. elegans*, model organisms with widely varying morphologies and size scales. While there exist a wide variety of prior algorithms reported in the literature, including recent reports using advanced machine-learning methods, ‘classic’ methods such as watershed segmentation can still have substantial advantages in robustness and computational efficiency. In this report we describe an algorithm which combines a logical procession of classic morphologic operations, which is able to successfully segment cells without relying on machine learning methods or on measures of convexity that are typically applied in prior work on yeast segmentation, allowing our algorithm to successfully identify a wider range of organisms and mutant types than prior methods. The algorithm has been demonstrated utilizing bright-field images alone, with an additional algorithm incorporating fluorescent images from yeast when available to slightly increase robustness (as shown in Fig. S2). Both of the algorithms can reach precisions in determining cell morphology similar to manual image analysis. However, as with other image analysis methods, our algorithm contains a few caveats. While our method outperforms other recently published results, currently the algorithm's performance does not yield a 100% success rate in segmentation. The majority of cases where images were not successfully analyzed was due to issues of imperfect image quality, or where individual yeast were morphologically unusual, perhaps due to cell death or other factors. Poorly-focused images and ‘dirty’ slides will both confound our current algorithm and care must be taken to ensure proper alignment of the microscope prior to image acquisition. As shown in Fig. S7, the accuracy of the method critically depends on the focus. The best performance is achieved slightly below focus, where the contrast between the cell wall and the background is highest, with a range of acceptable foci spanning approximately 1–5 µm below focus using our relatively high NA microscope system. As demonstrated by the *C. elegans* results, our algorithm is tolerant to highly non-uniform illuminations. Adding calcofluor fluorescence images to the pipeline mitigated these concerns to some extent in yeasts, due to the higher contrast between cell wall fluorescence and the dark background. In some cases, particularly with the cdc25 strain, the highly structured cytoplasm of the cells yielded slight over-segmentation in a small minority of cells. Further, some cells were treated as part of the image background due to a lack of contrast between the cell wall and the background. Both of these concerns could be overcome through more complex imaging methods such as dark field or quantitative phase imaging. However, phase imaging methods may require modification to the algorithm. In the case of traditional Zernike phase contrast, where a uniform halo is observed around the cell, our algorithm may indeed work with simple parameter tuning. However, other phase contrast methods such as DIC, where the cell wall does not maintain a constant intensity value, but instead undergoes a contrast reversal, would require more substantial modification. More complex binarization approaches such as using the Hilbert transform (Kuijper and Heise, 2008) rather than simple intensity thresholding could overcome this challenge. However, for determining the mean morphology of each cell type, culture condition, etc. the results obtained from simple bright-field imaging are sufficient even without a 100% correct segmentation rate. This is demonstrated by the high concordance between the mean length values for fission yeast regardless using the bright-field only and bright-field plus fluorescence algorithms, despite the fact that adding fluorescence increases the segmentation accuracy. Thus, our algorithm balances performance with experimental complexity: providing reasonable segmentation accuracy and highly accurate morphology measures using only bright-field imaging, with no need for exotic or automated imaging systems. Additionally, as our goal is to reduce manual effort as much as possible, all images examined for a given model organism have been processed using a single set of parameters in the image analysis pipeline. However, performance could be further optimized by altering the free parameters in our provided software on a strain-by-strain or even image-by-image basis, as dictated by the available time and requirements of the operator. Lastly, our algorithm is currently available as both a MATLAB code that can be freely edited by interested users, and as a stand-alone executable for users without MATLAB. As our method makes use of standard morphological processing, the algorithm presented here could potentially be transformed into a CellProfiler pipeline for those who wish to have more granular control of the algorithm but who are not experts in MATLAB programming.

## MATERIALS AND METHODS

### Strains and media

Fission yeast strains were created by random spore digestion as previously described (Forsburg and Rhind, 2006). The strain used for Calcofluor-White staining in Fig. 1 was wild type (ade6-m210 leu1-32 ura4-D18 h−). For Fig. 3, the strains used were as follows: wild type (ade6-m210 leu1-32 ura4-D18 h−), orb6-25 (ade6-m210 leu1-32 h−), tea1Δ (ade6-m210 leu1-32 h+), wee1Δ (leu1-32 h−), cdc25-22 (h+); the temperature sensitive strains orb6-25 and cdc25-22 were cultured first at the permissive temperature 30°C and then at the restrictive temperature 37°C for 4 h. For Fig. 6, the strains used were as follows: wild type (h−), tor2ts (h−), and igo1Δ (h+). Generally, fission yeast strains were cultured either in the rich medium YE5S (yeast extract medium supplemented with adenine, histidine, lysine, leucine, and Uracil) (www.formedium.com, Formedium Ltd. Hunstanton, Norfolk, UK) or in the defined medium EMM5S (Edinburgh minimal medium with adenine, histidine, lysine, leucine, and Uracil) at 30°C. For starvation, cells were grown in EMM without nitrogen (EMM-N). The tor2ts cells and their wild-type control cells in the exponential phase were collected and cultured in EMM-N for starvation and in YE5S for replenishment at the restrictive temperature 37°C. For budding yeast imaging, Y2H gold cells were cultured in YPDA medium at 30°C, and cells in the exponential phase were collected for imaging. The *C. elegans* worms were grown on agar plates containing *Escherichia coli* and sodium azide (to immobilize the worms).

### Starvation and release methods

The strains used for the starvation and release experiments were inoculated into YE5S medium at 30°C, and cells at the exponential phase were collected for imaging analysis and measurements. Briefly, cells were washed with EMM-N five times after collection. For nitrogen starvation analysis, the washed cells were cultured in EMM-N medium for 24 h; for replenishment analysis, the starved cells were then spun down and cultured in the rich medium YE5S. Images were taken during the starvation and replenishment at the time points indicated in the figures.

### Fluorescence staining and microscopic imaging

To visualize fission yeast cells with high contrast, Calcofluor-White staining was performed. Briefly, cells grown in YE5S medium were collected and washed with the PBS (phosphate buffered saline) buffer one time, followed by incubation in PBS containing 0.5 µg/ml Calcofluor-White (Sigma-Aldrich) in the dark for 10 min. The stained cells were then washed with PBS four times before imaging. Bright-field and fluorescence imaging was performed with an upright Olympus BX53 microscope (Olympus Corp., Tokyo, Japan) equipped with a condenser (U-AC2, NA1.1) and a 60x, NA 1.35 oil objective. Both bright-field and fluorescence images were acquired with a CCD camera (Olympus DP73). Similarly, the budding yeast cells were imaged with the Olympus BX53 microscope. For *C. elegans* imaging, the worms were placed on an agar pad on a slide and images were acquired with the Leica DFC310 FX microscope (Leica Microsystems, Wetzlar, Germany).

### Data analysis

All of the data was analyzed, using in-house scripts and functions written in MATLAB (R2017b, The Mathworks, Natick, MA, USA). And in order to benchmark our algorithm, we compared its performance to two previously published methods for yeast cell segmentation, PombeX and CellDetect, using two datasets. Dataset A was a subset of the images analyzed by our method, showing yeast with varying morphologies. Dataset B was obtained from Peng et al. (2013) and includes ten bright-field images.

Briefly, the previously published algorithms use machine learning to determine cell contours. While machine-learning methods can be quite accurate, their accuracy depends on the similarity between the training and test data. The PombeX algorithm uses bright-field images (and optional nuclear fluorescence images) to identify the cell membrane and cytoplasm. The algorithm then utilizes gradient vector flow snake to obtain cell contours, then uses a machine learning-based validation of cell contours to remove incorrect contours. The CellDetect algorithm takes simply annotated images (images paired with x-y coordinates of cell centroids) and uses these to train a structured SVM framework. In testing images a maximally stable extremal region detector is used to roughly dot annotate different cells, followed by using the trained SVM to evaluate and identify the non-overlapping regions as correct cells.

All the three test algorithms were run on a standard mid-range desktop computer, with an Intel i5-3470, 3.5 GHz CPU and 8GB RAM. As the magnification, resolution, and focus values of Dataset A and B differed, in order to provide the fairest comparison, separate classifiers were trained for Dataset A and Dataset B for CellDetect, and morphological parameters, particularly the binarization threshold, were re-optimized for our algorithm, as Datasets A and B had differing brightnesses. PombeX, meanwhile, had no user-controllable parameters, leading to fragile performance when applied to images outside of its original dataset.

## Supplementary Material

Supplementary information
