# Supplementary material for: Automated morphometry toolbox for analysis of microscopic model organisms using simple bright-field imaging
Source: Biol Open. 2019 Feb 27;8(3):bio037788. doi: 10.1242/bio.037788 (PMC6451328; doi:10.1242/bio.037788)
Supplement: Supplementary information [file biolopen-8-037788-s1.pdf]

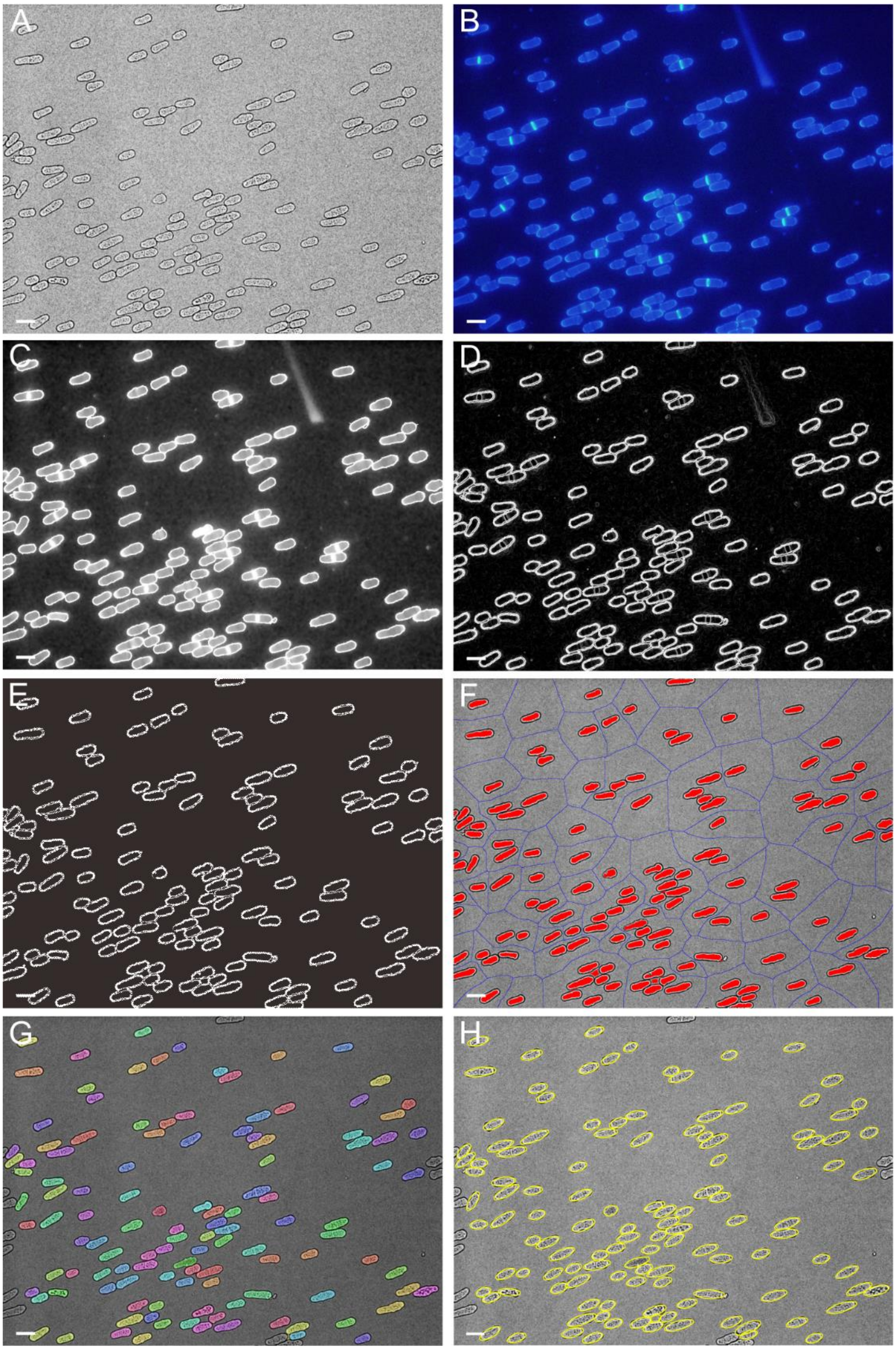

Figure S1. Graphical depiction of bright field and fluorescent (BF+Fluor) image combined algorithm. (A) Representative contrast-adjusted blue channel bright field image of wild-type (WT) fission yeast cells, scale bar corresponds to 10 microns. (B) Corresponding calcofluor white fluorescent image. (C) Combined image obtained by adding the cell wall mask (Figure S1C) and the green channel of the fluorescent image after it is denoised by a total-variation denoising algorithm. (D) Prewitt gradient of the combined image. (E) The final cell wall mask, obtained through binarizing the gradient image using Otsu's method, followed by morphologic opening and closing. (F) Image shown in (A) overlayed by foreground markers shown in red

(obtained through a logical AND operation between an inverted version of E and a version of E with holes filled) and background markers shown in blue produced through skeleton by influence zones on the cell wall mask. (G) Final segmented result, where a false color overlay has been added to identify segmented regions. (H) The contrast adjusted bright field image overlayed with ellipses shown in yellow, from which cell lengths and widths are extracted.

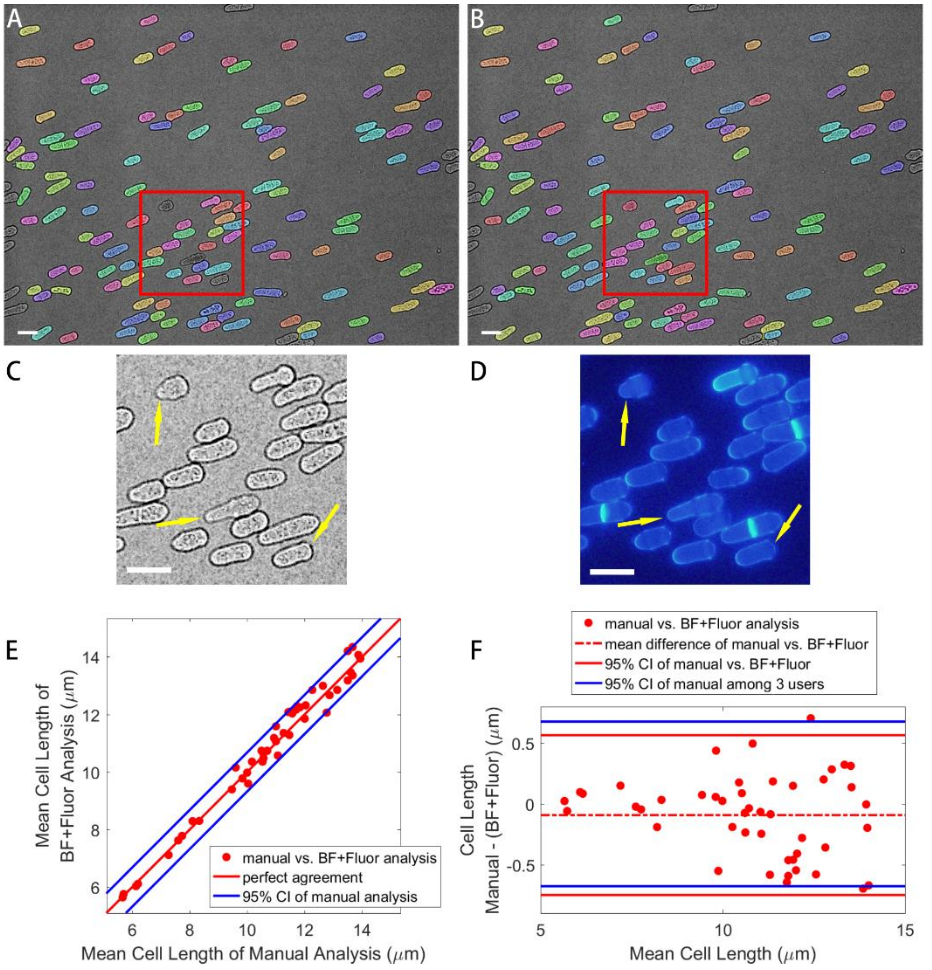

Figure S2. Segmentation comparison between BF only and BF+Fluor algorithms, and analysis results comparison between manual and BF+Fluor algorithm. (A) The final segmentation result of BF only algorithm, three cells where portions of the cell wall had lower contrast were lost, shown in the red square, scale bar corresponds to 10 microns. (B) The final segmentation result of BF+Fluor algorithm, since the fluorescent image has a higher contrast with background than bright field image, the algorithm can recognize more cells, as shown in the red square. (C) Magnified bright field image corresponding to the region of interest marked in A; the yellow arrows indicate the cell wall regions with low contrast. (D) Magnified fluorescence image corresponding to the region of interest marked in A; the yellow arrows indicate improved cell wall contrast. (E) Mean cell length comparison between manual and BF+Fluor analysis, each dot represents mean cell length within one of 46 images analyzed by manual vs. BF+Fluor analysis, blue lines represent the 95% confidence? Interval (CI) of manually-determined mean cell lengths among 3 different users. (F) Bland-Altman comparison between manual and BF+Fluor analysis, each dot represents the mean cell length within a single image.

Biology Open • Supplementary information

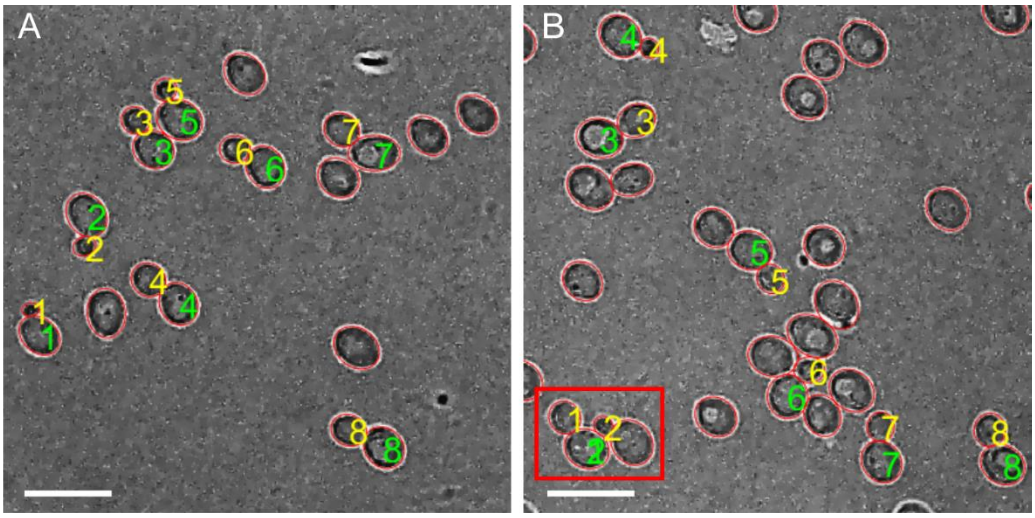

Figure S3. Examples of correctly and incorrectly paired parent and daughter budding yeast, scale bar corresponds to 10 microns. (A) The correctly paired parent and daughter cells marked by green and yellow text respectively. (B) The incorrectly paired parent and daughter cell highlighted in red rectangle, as the buds are closer to other mature cells than to their parent cells.

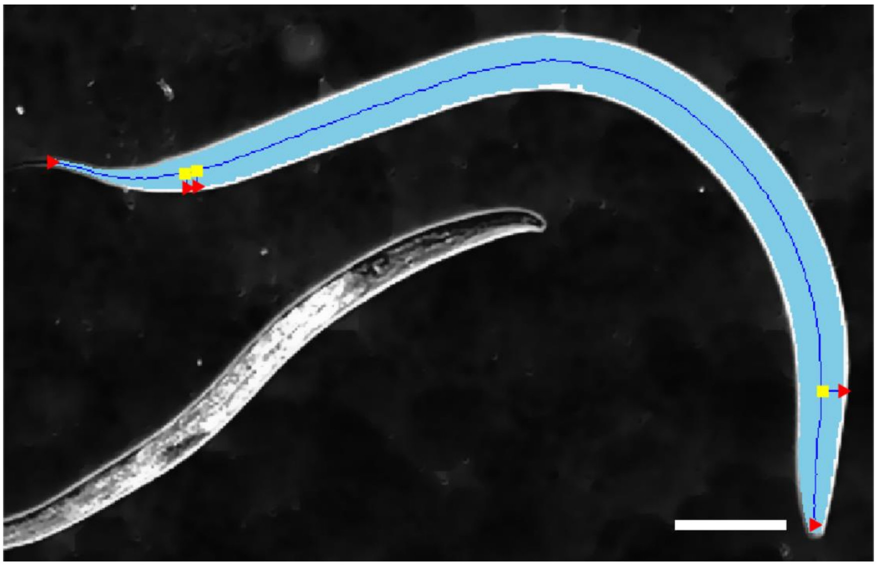

Figure S4. Automated length determination of *C. elegans*, scale bar corresponds to 50 microns. The final segmentation result is shown in sky blue, while the skeletonization of the worm is shown as a thin blue line. Yellow squares indicate branch points, while red triangles indicate endpoints.

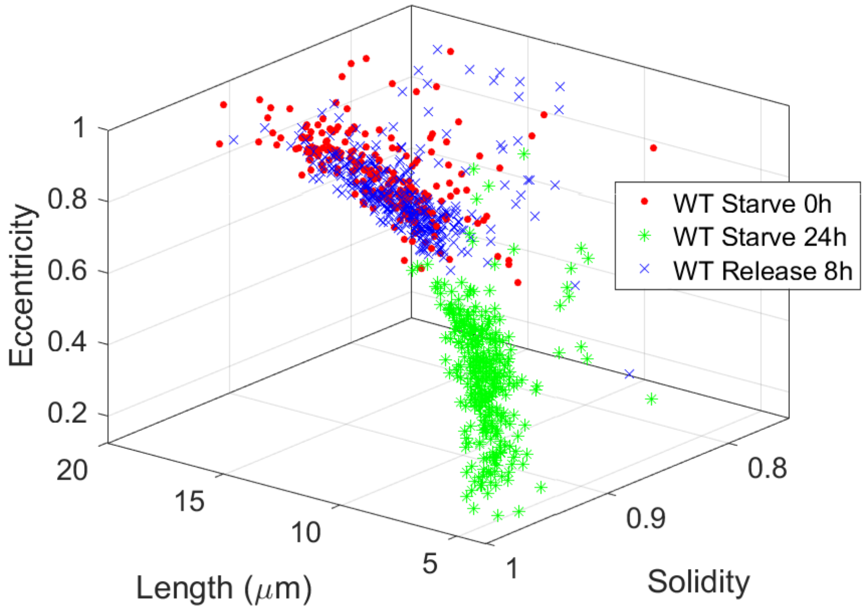

Figure S5. Morphologic analysis of nitrogen starvation and replenishment of wild type (WT) strain. Each dot represents morphologic parameters of one cell in the bright field images captured at starve 0 hours, starve 24 hours or release 8 hours. The cells in initially have a relatively longer length, higher eccentricity, with a somewhat broad range of solidity values; the cells after 24 hours of nitrogen starvation are small and round, corresponding to a shorter length, lower eccentricity, and stable solidity range reflecting their convex shapes; 8 hours after nitrogen replenishment, the cells have nearly the same distribution as in the initial condition.

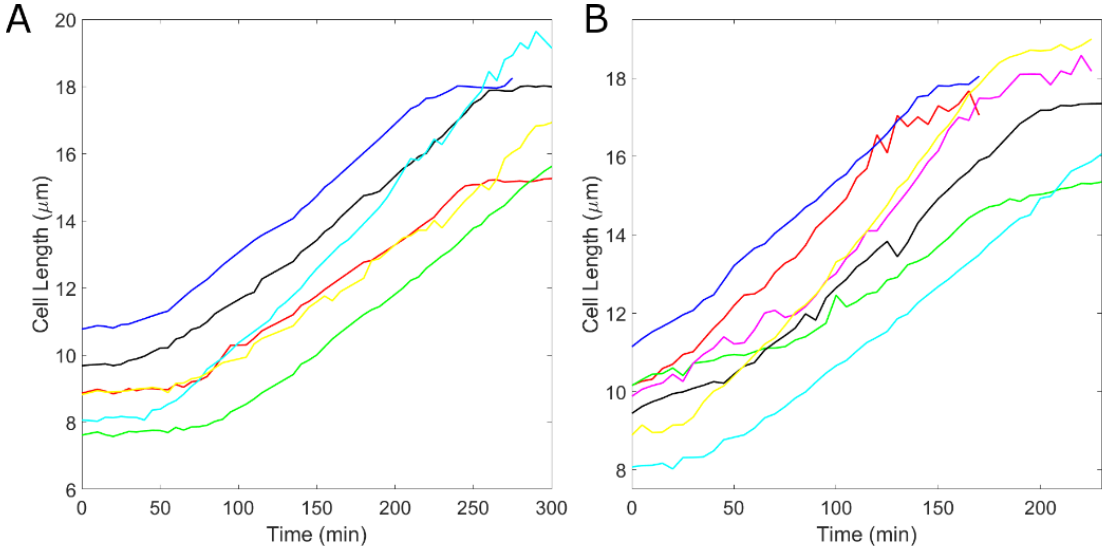

Figure S6. Single-cell time-lapse curves extracted from automatically segmented cells for (A) wild type S. pombe, and (B) tea1-deletion S. pombe. Cell growth curves are measured from division-to-division.

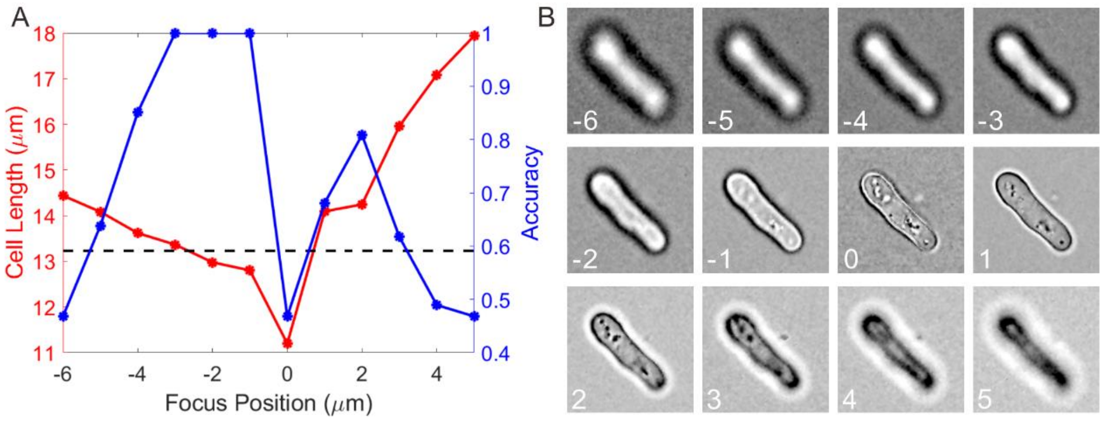

Figure S7. Performance of algorithm versus image quality, computed on a z-stack of S. pombe. (A) Comparison of segmentation accuracy (in blue) versus focal position, alongside extracted cell length (in red) compared with manually determined cell length (shown as a black dashed line). (B) ROI of a single yeast showing contrast changes versus defocus. Note that the optimum segmentation is observed when the image is slightly defocused, corresponding to large contrast reversal (bright-dark-bright) between the cell and the background.

Table S1. Correlation of mean cell length analyzed by various methods.

Group1	Group2	Correlation
User 1	User 2	0.9778
User 1	User 3	0.9764
User 2	User 3	0.9814
Mean Manual	BF only	0.9916
Mean Manual	BF + Fluor	0.9898

Table S2. Student’s t-test of length, solidity and eccentricity between WT and the other four strains.

Morphometric Parameter	Variable 1	Variable 2	P Value	Confidence Interval	
Length	WT	*orb6-25*	<0.001	2.0727	2.7048
		tea1 Δ	<0.01	-0.9103	-0.1383
		wee1 Δ	<0.001	2.1055	2.7403
		cdc25-22	<0.001	-.27.5172	-24.0135
Solidity	WT	*orb6-25*	<0.001	-0.0337	-0.0241
		tea1 Δ	<0.001	0.0219	0.0351
		wee1 Δ	<0.001	-0.0144	-0.0050
		cdc25-22	<0.001	0.0788	0.1001
Eccentricity	WT	*orb6-25*	<0.001	0.1942	0.2185
		tea1 Δ	0.356	-0.0037	0.0104
		wee1 Δ	<0.001	0.0830	0.1050
		cdc25-22	<0.001	-0.0793	-0.0675
